# Labor epidural analgesia and subsequent risk of offspring autism spectrum disorder and attention-deficit/hyperactivity disorder: a cross-national cohort study of 4.5 million individuals and their siblings

**DOI:** 10.1016/j.ajog.2022.08.016

**Published:** 2022-08-13

**Authors:** Tor-Arne Hegvik, Kari Klungsøyr, Ralf Kuja-Halkola, Hanna Remes, Jan Haavik, Brian M. D’Onofrio, Niina Metsä-Simola, Anders Engeland, Seena Fazel, Paul Lichtenstein, Pekka Martikainen, Henrik Larsson, Amir Sariaslan

**Affiliations:** Department of Biomedicine, University of Bergen, Bergen, Norway; Department of Heart Disease, Haukeland University Hospital, Bergen, Norway; Department of Global Public Health and Primary Care, University of Bergen, Bergen, Norway; Division of Mental and Physical Health, Norwegian Institute of Public Health, Bergen, Norway; Department of Medical Epidemiology and Biostatistics, Karolinska Institutet, Stockholm, Sweden; Population Research Unit, Faculty of Social Sciences, University of Helsinki, Helsinki, Finland; Department of Biomedicine, University of Bergen, Bergen, Norway; Bergen Center for Brain Plasticity, Division of Psychiatry, Haukeland University Hospital, Bergen, Norway; Department of Psychological and Brain Sciences, Indiana University, Bloomington, IA; Population Research Unit, Faculty of Social Sciences, University of Helsinki, Helsinki, Finland; Department of Global Public Health and Primary Care, University of Bergen, Bergen, Norway; Division of Mental and Physical Health, Norwegian Institute of Public Health, Bergen, Norway; Department of Psychiatry, University of Oxford, Warneford Hospital, Oxford, United Kingdom; Department of Medical Epidemiology and Biostatistics, Karolinska Institutet, Stockholm, Sweden; Population Research Unit, Faculty of Social Sciences, University of Helsinki, Helsinki, Finland; Centre for Health Equity Studies (CHESS), Stockholm University and Karolinska Institutet, Stockholm, Sweden; Max Planck Institute for Demographic Research, Rostock, Germany; Department of Medical Epidemiology and Biostatistics, Karolinska Institutet, Stockholm, Sweden; School of Medical Sciences, örebro University, örebro, Sweden; Department of Psychiatry, University of Oxford, Warneford Hospital, Oxford, United Kingdom

**Keywords:** attention-deficit/hyperactivity disorder, autism spectrum disorder, causal inference, family-based designs, labor epidural analgesia

## Abstract

**Background:**

A recent study has suggested that labor epidural analgesia may be associated with increased rates of offspring autism spectrum disorder. Subsequent replication attempts have lacked sufficient power to confidently exclude the possibility of a small effect, and the causal nature of this association remains unknown.

**Objective:**

This study aimed to investigate the extent to which exposure to labor epidural analgesia is associated with offspring autism spectrum disorder and attention-deficit/hyperactivity disorder following adjustments for unmeasured familial confounding.

**Study Design:**

We identified 4,498,462 singletons and their parents using the Medical Birth Registers in Finland (cohorts born from 1987e2005), Norway (1999e2015), and Sweden (1987e2011) linked with population and patient registries. These cohorts were followed from birth until they either had the outcomes of interest, emigrated, died, or reached the end of the follow-up (at mean ages 13.6–16.8 years), whichever occurred first. Cox regression models were used to estimate country-specific associations between labor epidural analgesia recorded at birth and outcomes (eg, at least 1 secondary care diagnosis of autism spectrum disorder and attention-deficit/hyperactivity disorder or at least 1 dispensed prescription of medication used for the treatment of attention-deficit/hyperactivity disorder). The models were adjusted for sex, birth year, birth order, and unmeasured familial confounders via sibling comparisons. Pooled estimates across all the 3 countries were estimated using inverse variance weighted fixed-effects meta-analysis models.

**Results:**

A total of 4,498,462 individuals (48.7% female) were included, 1,091,846 (24.3%) of which were exposed to labor epidural analgesia. Of these, 1.2% were diagnosed with autism spectrum disorder and 4.0% with attention-deficit/hyperactivity disorder. On the population level, pooled estimates showed that labor epidural analgesia was associated with increased risk of offspring autism spectrum disorder (adjusted hazard ratio, 1.12; 95% confidence interval, 1.10–1.14, absolute risks, 1.20% vs 1.07%) and attention-deficit/hyperactivity disorder (adjusted hazard ratio, 1.20; 95% confidence interval, 1.19–1.21; absolute risks, 3.95% vs 3.32%). However, when comparing full siblings who were differentially exposed to labor epidural analgesia, the associations were fully attenuated for both conditions with narrow confidence intervals (adjusted hazard ratio [autism spectrum disorder], 0.98; 95% confidence interval, 0.93–1.03; adjusted hazard ratio attention-deficit/hyperactivity disorder, 0.99; 95% confidence interval, 0.96–1.02).

**Conclusion:**

In this large cross-national study, we found no support for the hypothesis that exposure to labor epidural analgesia causes either offspring autism spectrum disorder or attention-deficit/hyperactivity disorder.

## Introduction

Labor epidural analgesia is commonly used worldwide to provide pain relief to women in labor, as it is effective and considered safe.^1–4^ The most common side effects for mothers are typically temporary and relatively mild (eg, urinary retention and maternal fever),^1,2^ and more serious side effects (eg, epidural hematoma and deep infections) remain extremely rare.^5,6^ However, a few studies have examined long-term outcomes in offspring exposed to labor epidural analgesia.^2^ A recent cohort study^7^ of nearly 148,000 children in the United States reported that labor epidural analgesia may be associated with an up to 37% increased risk of offspring autism spectrum disorder (ASD). Criticism of the study, including from several medical societies,^8–10^ have raised concerns about the methodologic limitations of the study, the lack of biological plausibility of the proposed association, and the possible clinical implications that an implied causal inference might have.^11–13^

A key limitation of this and other observational studies is that unmeasured genetic confounders that may simultaneously increase the likelihood of the exposure (ie, labor epidural analgesia) and the outcome (ie, offspring ASD) have not been adequately accounted for. This is important for 2 reasons. First, it has been demonstrated that women who elect to give birth using labor epidural analgesia tend to have elevated anxiety and depressive symptoms,^14,15^ which are moderately heritable traits.^16,17^ Second, twin and family-based pedigree studies have consistently found that genetic influences account for approximately 80% of the individual risk differences in ASD,^18–20^ which partly overlap with those explaining individual differences in anxiety and depressive symptoms.^21–23^ It has therefore been recommended that investigations of possible pre- and perinatal risk factors for neurodevelopmental disorders should adopt family-based research designs to account for unmeasured familial confounding (eg, genetic and early-life environmental risks shared within families).^24^

To our knowledge, there are currently 4 published replication attempts of the original study,^7^ which have used population-based data from Canada^25,26^ and Denmark,^27,28^ in combination with the genetically informative ‘within-mother’ design; here, the risks of offspring ASD were compared between maternal siblings who were differentially exposed to labor epidural analgesia.^29^ This approach allowed the researchers to account for a portion of the genetic differences shared between siblings.^30^ Although these studies have consistently demonstrated that population-wide associations between labor epidural analgesia and offspring ASD (odds/hazard ratio range, 1.05–1.32) were fully attenuated in the adjusted within-mother models, they have lacked sufficient statistical power to confidently exclude the possibility of a small and potentially causal association.

To address these limitations in previous research and assess the long-term safety of labor epidural analgesia in relation to ASD risk, we used nationwide register data from 3 Nordic countries (Finland, Norway, and Sweden) to examine the associations between labor epidural analgesia on the subsequent risk of offspring ASD across 4.5 million singletons. On the basis of the previous replication studies,^25–28^ we hypothesized that this association would be fully explained by unmeasured familial confounders. We also investigated attention-deficit/hyperactivity disorder (ADHD) as an outcome, as it is a more prevalent neurodevelopmental disorder that shares some of its genetic etiology with ASD.^21,31,32^ Importantly, to account for unmeasured familial confounders, we combined statistical methods that accounted for measured covariates with a research design comparing the outcome rates between biological full siblings who were differentially exposed to labor epidural analgesia. We were able to estimate these associations with greater precision than in previous studies by pooling associations from all 3 countries and weighing them according to their population sizes using meta-analytical models.

## Materials and Methods

### Study population

We linked several Nordic nationwide population registry data to generate country-specific samples. All residents in Nordic countries are assigned a personal identification number, which is used in respective nationwide registers and provides accurate linkage.^33^ We were granted permission to use pseudonymized data following approvals from the Ethics Board of Statistics Finland (TK-53-1121-18), the Regional Committees for Medical Research Ethics in Norway (2020/75421), and the Regional Ethical Review Board in Stockholm, Sweden (2013/862-31/5). We conducted the data analyses separately on secure servers located in each country. The output, which included the magnitude of the associations and their uncertainties (ie, standard errors), was then used as input data for a metaanalytical model that estimated the pooled associations across all 3 countries while accounting for their population size differences. Informed consent is not required for register-based studies in the Nordic countries.

We initially identified all singleton children born in Finland from 1987 to 2005 (n = 1,125,424), Norway (1999–2015) (n=965,882), and Sweden (1987–2011) (n = 2,512,569) using the population-wide Medical Birth Registers in each country,^34^ which also provided data on labor epidural analgesia use, offspring gestational age, mode of delivery, and maternal age at delivery. The sample thus included a combined total of 4,603,875 children. We then prospectively identified individuals who had ever been diagnosed with ASD and ADHD in the Finnish Care Register for Health Care, which included all inpatient care episodes from 1987 to 2017 and specialist outpatient care visits from 1998 to 2017 according to the Ninth and Tenth revisions of the International Classification of Diseases (ICD-9 and ICD-10).^35^ We similarly identified the same patient groups in the Norwegian Patient Register (inpatient and outpatient care 2008–19, ICD-10)^36^ and the Swedish Patient Register (inpatient care: 1987–2013; outpatient care: 2001–13, ICD-9 and ICD-10).^37^ Data on ADHD medications were gathered from the prescribed drug registers in Finland (1995–2018), Norway (2004–2019), and Sweden (2005–2014).^38^ Migration and mortality dates were retrieved from the population registers in Finland and Norway and the Migration and Causes of Death registers in Sweden. The average age at the end of follow-up was 15.9 years.

We constructed our analytical sample by excluding individuals who could not be linked to both of their biological parents (n = 56,846 [1.2%]) and had missing data on gestational age at birth (n = 14,846 [0.3%]) and their mode of delivery (n = 897 [0.02%]). We further removed those who had either migrated (n = 21,383 [0.5%]) or died (n=11,441 [0.3%]) before reaching their first birthday in Finland and Sweden and before their fifth birthday in Norway (owing to the outcome data being available at a later date). Our analytical sample consequently retained 97.7% of the targeted sample (n=4,498,462). Country-specific sample sizes are provided in the Supplemental Table.

### Exposures and outcomes

Labor epidural analgesia was defined as a binary measure derived from the Medical Birth Registries in each country, where midwives attending birth had recorded the type of analgesia, if any, that the mothers had received in labor. No data were available on the solution types or dosages used.

We defined individuals who had been diagnosed with ASD (ICD-9: 299, ICD-10: F84) on at least 1 occasion to have ASD and identified the first observed diagnosis date.^19^ Similarly, we defined individuals who had received at least 1 diagnosis of ADHD (ICD-9: 314, ICD-10 F90) or who at least once dispensed a prescription of medications used nearly exclusively in the treatment of ADHD (Anatomical Therapeutic Chemical codes: N06BA01, N0BA02, N06BA04, N06BA09, N06BA12) to have ADHD and identified the first observed diagnosis date or date of first prescription. Single-episode diagnoses of ASD and ADHD in the national healthcare registers have been found to have excellent validity (ie, positive predictive values varying between 88% and 96%) across Finland,^39,40^ Norway,^41^ and Sweden.^42,43^

#### Analytical approach

We quantified the crude population-wide associations between labor epidural analgesia and subsequent risks for offspring ASD and ADHD, expressed as adjusted hazard ratios (aHRs), using Cox proportional hazards regression models. The underlying time scale was defined as time from birth to the first of any of the following events: having the outcome of interest, emigration, death, or reaching the end of the follow-up (End of follow-up Finland: December 31, 2017 for ASD and December 31, 2018 for ADHD; End of follow-up Norway: December 31, 2019; End of follow-up Sweden: December 31, 2013 for ASD and December 31, 2014 for ADHD). These models were adjusted for sex, birth year (each year as a separate category), and birth order (categorized into 1, 2, 3, and >4). To further account for the time-stable unmeasured familial confounding shared between full siblings (ie, their shared early-life environments and an average of half of their cosegregating genes),^30^ we fitted analogous stratified Cox regression models, which allowed for the baseline hazards to vary across families, thus implying that the risk comparisons were made within families and between differentially exposed full siblings.^30,44^ To increase the precision of the estimates, we subsequently pooled the country-specific estimates using the inverse variance weighted fixed-effects metaanalytic model, which weighs the estimates from each country by their relative sample size.^45^

In complementary sensitivity analyses, we excluded the offspring born by cesarean delivery and those born prematurely (gestational age <37 weeks) and defined individuals as having ASD or ADHD only if they had been diagnosed with each condition (or dispensed ADHD medications) at 2 separate instances. In addition, we examined ADHD as an outcome using only the patient data. The sibling comparison design assumes that the siblings are generalizable to the full population, and the absence of any birth order or carry-over effects, namely that the exposure and outcome of a given sibling in a family, do not influence the exposures and outcomes of their cosiblings. To test for these assumptions, we initially fitted the population-wide models on a subset of all siblings. We then reexamined the associations in a subset of all first-born cousins in Finland and Sweden who were differentially exposed to labor epidural analgesia (n=155,299).

## Results

**Figure 1:**
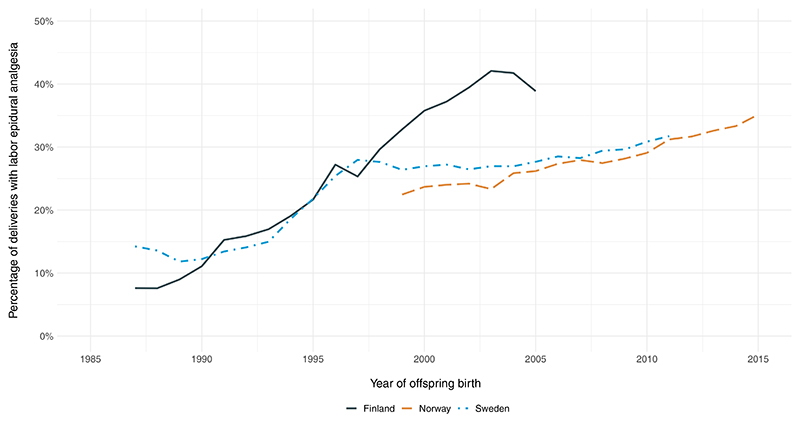
Percentage of births with labor epidural analgesia in Finland, Norway, and Sweden between 1987 and 2015

We examined a total of 4,498,462 individuals including those from Finland (n = 1,097,266), Norway (n = 929,560), and Sweden (n = 2,471,636) between 1987 and 2015, of which 1,091,846 (24.3%) were exposed to labor epidural analgesia (Table 1). There was considerable variation in the rates of labor epidural analgesia across time, ranging from approximately 10% at the baseline of the study to approximately 35% to 40% at the end of the follow-ups, with relatively small between-country differences (Figure 1). The crude absolute risks of the outcomes were marginally elevated among individuals who were exposed to labor epidural analgesia compared with unexposed individuals (ASD: 1.20% vs 1.07%; ADHD: 3.95% vs 3.32%) (Table 2).

**Figure 2:**
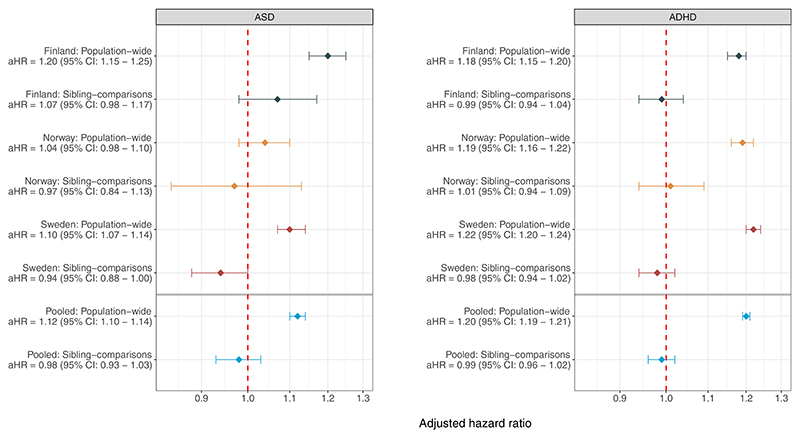
Associations between labor epidural analgesia and offspring ASD and ADHD. All models were adjusted for sex, birth year, and birth order. The sibling comparison models further accounted for unmeasured familial confounders shared between full siblings. *aHR*, adjusted hazard rates; *ADHD*, attention-deficit/hyperactivity disorder; *ASD*, autism spectrum disorder; *CI*, confidence interval. *Hegvik et al. Labor epidural analgesia and subsequent risk of offspring autism spectrum disorder and attention-deficit/hyperactivity disorder. Am J Obstet Gynecol 2023*.

After pooling estimates across all 3 countries, we found that labor epidural analgesia was associated with an approximately 12% increased risk of ASD (adjusted hazard ratio [aHR], 1.12; 95% confidence interval [CI], 1.10–1.14) and a 20% increased risk of ADHD (aHR, 1.20; 95% CI, 1.19–1.21) following adjustments for sex, birth year, and birth order (Figure 2). To further account for shared, unmeasured familial confounders (eg, genetic risks and early-life environmental factors), we subsequently compared the hazards of the outcomes between 985,444 full siblings who were differentially exposed to labor epidural analgesia, of which 24,516 had later developed ASD and 68,991 developed ADHD (Table 3). We found that those exposed to labor epidural analgesia were no more likely than their unexposed siblings to be diagnosed with either ASD (aHR, 0.98; 95% CI, 0.93–1.03) or ADHD (aHR, 0.99; 95% CI, 0.96–1.02) (Figure 2).

In the complementary sensitivity analyses, we obtained similar estimates as in the main sibling comparison models when we excluded cesarean deliveries (aHR_ASD_, 1.02; 95% CI, 0.98–1.04). We further found commensurate results when we used stricter definitions requiring at least 2 diagnoses or ADHD medication purchases on separate occasions (aHR_ASD_, 1.01; 95% CI, 0.95–1.07; aHR_ADHD_, 1.00; 95% CI, 0.97–1.03) or by excluding ADHD medication purchases (aHR_ADHD_, 0.98; 95% CI, 0.94–1.02). To test for the generalizability of the sibling comparison estimates, we initially ran the population-wide models in the sibling subsets and found similar results (aHR_ASD_, 1.12; 95% CI, 1.09–1.15; aHR_ADHD_, 1.20; 95% CI, 1.18–1.22). We subsequently tested for the potential impact of carry-over effects by examining within-extended family associations using first-born cousins who were differentially exposed to labor epidural analgesia. The within-extended family association between labor epidural analgesia and ASD was completely attenuated (aHR, 1.02; 95% CI, 0.92–1.12), and the equivalent association with ADHD was attenuated by 35% (aHR, 1.13; 95% CI, 1.06–1.20).

## Comment

### Principal findings

In our cohort study of 4.5 million singleton births in Finland, Norway, and Sweden, including over 985,000 siblings who were differentially exposed to labor epidural analgesia, we did not find support for the hypothesis that labor epidural analgesia causes subsequent increased risks of offspring ASD or ADHD.

### Results in context

First, in our initial population-wide analyses, we found that labor epidural analgesia was associated with a 12% increased risk of developing ASD and a 20% increased risk of developing ADHD. These results are broadly consistent with earlier reports focusing on the population-wide association between labor epidural analgesia and offspring ASD in Denmark,^27,28^ Canada,^25,26^ and the United States,^7^ though the magnitude of the reported estimates have varied widely across studies (odds/hazard ratio range, 1.05–1.37). Although these variations could arise from country and temporal factors, they could be attributed to different methods (eg, using administrative register data vs private health insurance data, matching procedures, and statistical model selection).

Second, we found that the associations between labor epidural analgesia and offspring risks of ASD and ADHD were entirely attenuated once we accounted for unmeasured familial confounders (ie, genetic and early-life environmental influences) shared between biological full siblings who were differentially exposed to labor epidural analgesia. In what is inconsistent with a causal interpretation, we found that the siblings who were exposed to labor epidural analgesia were no more likely than their unexposed cosiblings to develop either ASD or ADHD. These findings could not be attributed to limited within-family variation in either the exposure or the outcomes, the inclusion of cesarean deliveries and those born preterm, or stricter outcome definitions.

These findings are in keeping with recent population-based Canadian^25,26^ and Danish^27,28^ studies that used a within-mother design to examine the association between labor epidural analgesia and ASD while accounting for unmeasured familial confounding. The earlier work had primarily stratified their models across clusters of mothers instead of both biological parents, which implies that their sibling comparisons included a combination of both full siblings and maternal half-siblings. Such an approach boosts the statistical power by increasing the number of differentially exposed siblings, but this comes with the limitation of lower internal validity (ie, poorer adjustments for un-measured genetic confounding, as maternal half-siblings share, on average, a quarter of their cosegregating genes). Although this earlier work did not find that siblings who were exposed to epidural analgesia during labor were more likely than their unexposed siblings to develop ASD, estimates lacked statistical power, which resulted in wide CIs. These previous studies could therefore not exclude the possibility of a moderately sized association, given that upper bounds of the confidence intervals were consistent with a risk increase ranging between 21%^25^ and 31%.^26^

Our third main finding is that our pooled sibling comparison estimate of the same labor epidural analgesia-ASD association, which was based on a nearly 3-fold larger population sample than the previous studies combined, enabled us to exclude the possibility of nonprecise estimation; this was because the upper bound of its confidence interval was consistent with a negligible maximum risk increase of 3%. Our study therefore adds to the accumulated evidence that does not support a causal interpretation of the associations between labor epidural analgesia with offspring neurodevelopmental disorders. Moreover, our findings are consistent with the literature reporting that a broader set of pre- and perinatal risk markers (eg, cesarean deliveries,^46^ labor induction,^47,48^ maternal infections,^49^ and smoking during pregnancy^50–52^) are not associated with offspring neu-rodevelopmental disorders once unmeasured familial confounders have been adequately accounted for.

### Clinical implications

Our findings suggest that the recommendation in the current clinical guidelines^53–55^ of informing and complying with the requests of pregnant women for labor epidural analgesia does not need to be revised, as we have demonstrated that the risks of offspring developing ASD or ADHD as a result of such exposure are negligible, if anything, and they are not clinically significant.

### Research implications

Our findings further demonstrate that though sibling comparison designs are effective in accounting for unmeasured familial confounding, they frequently require very large sample sizes to be sufficiently powered to inform null results. The latter is because the design is driven by the number of siblings who are differentially exposed to the risk factor of interest and who have different outcomes than their cosiblings.^30^ Therefore, if either the risk factor or the outcomes are uncommon in a given study, the sibling comparisons will be based on much smaller sample sizes. In the present study, we had 3 population samples totaling 4.5 million individuals, but our effective sibling sample sizes (ie, siblings who were differentially exposed to labor epidural analgesia and had different neurodevelopmental outcomes) were <2% of that (n_ASD_ = 24,516; n_ADHD_ = 68,991). We have shown that it is possible for studies in reproductive epidemiology to harmonize data across different population registers into a common pipeline and pool the associations using metaanalytical techniques to improve the statistical power of the analyses; this increases the precision of the estimates. International collaborations of this nature may therefore be necessary in the future to address etiological research questions with significant clinical implications requiring very large sample sizes.

### Strengths and limitations

Our study had several important strengths. The combination of nation-wide registry data from 3 Nordic countries, all with universal and accessible high-quality healthcare, enabled us to study 4.5 million individuals while keeping selection biases to a minimum. We were able to test for the association between labor epidural analgesia and externally validated diagnoses of ASD in the offspring. In addition, we considered offspring ADHD as an outcome owing to its clinical significance, common etiology with ASD, and higher prevalence.^31,32^. The inclusion of both conditions contributed to an improvement of the generalizability of our findings. Importantly, we adopted the sibling comparison design to account for unmeasured familial confounding between biological full siblings who share their early-life environmental influences and an average of half of their cosegregating genes. The consistency of the risk estimates between the 3 countries further added to the generalizability of our findings.

Our study had some important limitations. First, we did not have access to outpatient care and prescription drug data across the entire follow-up period for the older cohorts. In addition, we did not have access to inpatient care data in Norway before 2008. We may have consequently overestimated the age at which the conditions were first identified in the older cohorts. However, despite these differences in data availability, the findings remained similar across all 3 countries, and in the case of ADHD, we also found commensurate results when we excluded medication data. Second, the sibling comparison design requires very large sample sizes and assumes that the siblings are generalizable to the general population and that no birth order or sibling carry-over effects exist (eg, exposure of labor epidural analgesia in older siblings affecting the exposure and outcome in their younger cosiblings).^56^ We rigorously tested for these assumptions in complementary sensitivity analyses and did not find any evidence that they were violated. Third, though the sibling comparison design is an effective method to account for time-stable unmeasured familial confounders shared between siblings, it requires that nonshared confounders are included as measured covariates in the statistical models.^30^ In the present study, we adjusted for a subset of potential non-shared confounders (ie, gender, birth year, and birth order), but not all. For instance, because of being born in different years, some of the siblings were exposed to different early-life environmental risks compared with their cosiblings, including relative family income poverty^57,58^ and residing in socioeconomically deprived neighborhoods.^59^ Parental separation may have further caused some of the siblings to grow up in different households. However, it is unlikely that the inclusion of the latter nonshared confounders would have altered our findings, as the sibling comparison models yielded null results, indicating that there were no residual associations left to be explained by the adjustment for additional confounders. Fourth, we were unable to assess any potential dosage-response effects, as we did not have access to data on the duration of labor epidural analgesia exposure. Although this remains an empirical question that could be addressed in future studies, a recent nationwide Danish study^28^ was not able to replicate the dosage-response pattern of previously reported associations.^7^

In relation to generalizability, our estimated prevalence rates of ASD^60^ and ADHD^61^ were similar to the rates in other high-income countries. Labor epidural analgesia use increased considerably throughout the follow-up period across all the 3 countries that we examined with relatively small between-country differences, suggesting that the findings are generalizable across the Nordic countries. Given that our findings were consistent with within-mother associations in Canada^25,26^ where the rates of labor epidural analgesia use have been consistently high throughout the same time period, it suggests that our findings may also be generalizable to other high-income countries.

### Conclusions

In this cross-national cohort study examining the associations between labor epidural analgesia and the subsequent risks of offspring ASD and ADHD, we found that the associations were entirely attenuated once we accounted for unmeasured familial confounders shared between exposure-discordant full siblings. In contrast with previous sibling comparison studies examining these associations, we had sufficient statistical power to confidently exclude the possibility of a larger-than-negligible association. We conclude that it is unlikely that labor epidural analgesia causes an increased risk of either offspring ASD or ADHD. Pregnant women therefore have no reason to fear that their decision to use epidural analgesia during labor will have any meaningful impact on their offspring’s risk of developing neuro-developmental disorders such as ASD or ADHD.

## Supplementary Material

Supplemental Table

## Figures and Tables

**Table 1:** Baseline demographic characteristics

Characteristic		Finland (n = 1,097,266)			Norway (n = 929,560)			Sweden (n = 2,471,636)			Pooled (n = 4,498,462)	
		Unexposed to LEA (%)	Exposed to LEA (%)		Unexposed to LEA (%)	Exposed to LEA (%)		Unexposed to LEA (%)	Exposed to LEA (%)		Unexposed to LEA (%)	Exposed to LEA (%)
Total		829,526 (76.6)	267,740 (24.4)		670,362 (72.1)	259,198 (27.9)		1,906,728(77.1)	564,908 (22.9)		3,406,616(75.7)	1,091,846 (24.3)
Sex												
	Female	407,420 (49.1)	128,938 (48.2)		329,084 (49.1)	123,247 (47.5)		934,371 (49.0)	267,530 (47.4)		1,670,875 (49.0)	519,715 (47.6)
	Male	422,106 (50.9)	138,802 (51.8)		341,278 (50.9)	135,951 (52.5)		972,357 (51.0)	297,378 (52.6)		1,735,741 (51.0)	572,131 (52.4)
Birth year												
	1987–1989	162,751 (19.6)	14,295 (5.3)		—	—		274,463 (14.4)	41,631 (7.4)		437,214 (12.8)	55,926 (5.1)
	1990–1994	262,623 (31.7)	48,744 (18.2)		—	—		483,557 (25.4)	82,529 (14.6)		746,180 (21.9)	131,273 (12.0)
	1995–1999	206,376 (24.9)	77,136 (28.8)		43,057 (6.4)	12,475 (4.8)		321,423 (16.9)	111,164 (19.7)		570,856 (16.8)	200,775 (18.4)
	2000–2004	163,917 (19.8)	106,024 (39.6)		202,123 (30.2)	64,575 (24.9)		326,265 (17.1)	120,099 (21.3)		692,305 (20.3)	290,698 (26.6)
	2005–2009	33,859(4.1)	21,541 (8.0)		200,993 (30.0)	75,982 (29.4)		355,373 (18.6)	143,164 (25.3)		590,225 (17.3)	240,687 (22.0)
	2010–2014	—	—		189,376 (28.2)	87,293 (33.6)		145,647 (7.6)	66,321 (11.7)		335,023 (9.8)	153,614 (14.1)
	2015	—	—		34,813 (5.2)	18,873 (7.3)		—	—		34,813 (1.0)	18,873 (1.7)
Birth order												
	First	329,778 (39.8)	128,311 (47.9)		221,631 (33.1)	161,803 (62.4)		666,705 (35.0)	392,480 (69.5)		1,218,114(35.8)	682,594 (62.5)
	Second	279,418 (33.7)	92,320 (34.5)		268,228 (40.0)	68,201 (26.3)		769,382 (40.4)	128,147 (22.7)		1,317,028 (38.7)	288,668 (26.4)
	Third	136,290 (16.4)	33,027 (12.3)		127,765 (19.1)	21,578 (8.3)		326,346 (17.1)	33,094 (5.9)		590,401 (17.3)	87,699 (8.0)
	Fourth or higher	84,040 (10.1)	14,082 (5.3)		52,738 (7.9)	7616(2.9)		144,295 (7.6)	11,187 (2.0)		281,073 (8.3)	32,885 (3.0)
Mother born abroad												
	No	799,363 (96.4)	254,457 (95.0)		535,853 (79.9)	207,405 (80.0)		1,577,108(82.7)	472,723 (83.7)		2,912,324 (85.5)	934,585 (85.6)
	Yes	30,163 (3.6)	13,283 (5.0)		124,724 (18.6)	48,855 (18.8)		329,620 (17.3)	92,185 (16.3)		484,507 (14.2)	154,323 (14.1)
	Missing	—	—		9785 (1.5)	2938 (1.1)		—	—		9785 (0.3)	2938 (0.3)
Maternal age at delivery (y)												
	≤17	1752 (0.2)	1362 (0.5)		1999 (0.3)	1474 (0.6)		6112 (0.3)	3580 (0.6)		9863 (0.3)	6416 (0.6)
	18–19	10,365 (1.2)	7136 (2.7)		9229 (1.4)	6348 (2.4)		27,493 (1.4)	13,986 (2.5)		47,087 (1.4)	27,470 (2.5)
	20–24	117,093 (14.1)	53,392 (19.9)		87,975 (13.1)	45,800 (17.7)		303,664 (15.9)	112,526 (19.9)		508,732 (14.9)	211,718 (19.4)
	25–29	275,765 (33.2)	94,249 (35.2)		215,421 (32.1)	87,862 (33.9)		641,771 (33.7)	195,374 (34.6)		1,132,957 (33.3)	377,485 (34.6)
	30–34	262,769 (31.7)	73,961 (27.6)		229,982 (34.3)	79,430 (30.6)		602,510 (31.6)	163,932 (29.0)		1,095,261 (32.2)	317,323 (29.1)
	35–39	128,677 (15.5)	30,906 (11.5)		106,436 (15.9)	32,634 (12.6)		271,564 (14.2)	64,291 (11.4)		506,677(14.9)	127,831 (11.7)
	40–44	31,201 (3.8)	6404 (2.4)		18,556 (2.8)	5458 (2.1)		51,514(2.7)	10,854 (1.9)		101,271 (3.0)	22,716(2.1)
	≥45	1904(0.2)	330(0.1)		764(0.1)	192 (0.1)		2100(0.1)	365 (0.1)		4768(0.1)	887 (0.1)

*LEA, labor epidural analgesia*.

*Hegvik et al. Labor epidural analgesia and subsequent risk of offspring autism spectrum disorder and attention-deficit/hyperactivity disorder. Am J Obstet Gynecol 2023*.

**Table 2:** Person-time at risk, number of patients, prevalence rates, and incident rates per 1000 person-years for autism spectrum disorder and attention-deficit hyperactivity disorder stratified across individuals exposed to labor epidural analgesia

Country		LEA	Population size	Person-years at risk		Number of patients	Prevalence rate (95% CI)	Incidence rate per 1000 person-years (95% CI)
				Total	Mean (standard deviation)			
Finland									
	ASD							
		No	829,526 (75.6%)	18,409,039	22.2 (5.7)	8520	1.03% (1.01%―1.05%)	0.5 (0.5―0.5)
		Yes	267,740 (24.4%)	5,017,184	18.7(5.2)	3743	1.40% (1.35%―1.44%)	0.7 (0.7―0.8)
	ADHD							
		No	829,526 (75.6%)	1,906,4091	23.0 (6.0)	26,744	3.22% (3.19%―3.26%)	1.4 (1.4―1.4)
		Yes	267,740 (24.4%)	5,214,454	19.5 (5.5)	12,344	4.61% (4.53%―4.69%)	2.4 (2.3―2.4)
Norway								
	ASD							
		No	670,362 (72.1%)	8,130,884	12.1 (4.9)	4331	0.65% (0.63%―0.67%)	0.5 (0.5―0.5)
		Yes	259,198 (27.9%)	2,918,292	11.3 (4.9)	1813	0.70% (0.67%―0.73%)	0.6 (0.6―0.7)
	ADHD							
		No	670,362 (72.1%)	8,045,820	12.0(4.8)	21,802	3.25% (3.21%―3.30%)	2.7 (2.7―2.7)
		Yes	259,198 (27.9%)	2,883,688	11.1 (4.8)	9347	3.61% (3.53%―3.68%)	3.2 (3.2―3.3)
Sweden								
	ASD							
		No	1,906,728(77.1%)	28,507,860	15.0 (7.5)	23,751	1.25% (1.23%―1.26%)	0.8 (0.8―0.8)
		Yes	564,908 (22.9%)	69,727,41	12.3 (7.0)	7516	1.33% (1.30%―1.36%)	1.1 (1.1―1.1)
	ADHD							
		No	1,906,728(77.1%)	30,137,810	15.8 (7.5)	64,720	3.39% (3.37%―3.42%)	2.1 (2.1―2.2)
		Yes	564,908 (22.9%)	7,456,083	13.2 (7.0)	21,397	3.79% (3.74%―3.84%)	2.9 (2.8―2.9)
Pooled								
	ASD							
		No	3,406,616 (75.7%)	55,047,783	16.2 (7.5)	36,602	1.07% (1.06%―1.09%)	0.7 (0.7―0.7)
		Yes	1,091,846 (24.3%)	14,908,217	13.6 (6.8)	13,072	1.20% (1.18%―1.22%)	0.9 (0.9―0.9)
	ADHD							
		No	3,406,616 (75.7%)	57,247,721	16.8 (7.7)	113,266	3.32% (3.31%―3.34%)	2.0 (2.0―2.0)
		Yes	1,091,846 (24.3%)	15,554,225	14.2 (6.9)	43,088	3.95% (3.91%―3.98%)	2.8 (2.7―2.8)

*ADHD*, attention-deficit/hyperactivity disorder; *ASD*, autism spectrum disorder; *CI*, confidence interval; *LEA*, labor epidural analgesia.

*Hegvik et al. Labor epidural analgesia and subsequent risk of offspring autism spectrum disorder and attention-deficit/hyperactivity disorder. Am J Obstet Gynecol 2023*.

**Table 3:** The number of siblings who were differentially exposed to labor epidural analgesia, had different outcomes, or were both differentially exposed and had different outcomes

Country – outcome		Number of siblings discordant on the following:		
		Labor epidural analgesia	Outcome	Both labor epidural analgesia and outcome
Finland				
	ASD	248,132	18,975	6609
	ADHD	248,132	54,502	19,521
Norway				
	ASD	205,053	8080	2698
	ADHD	205,053	35,379	12,424
Sweden				
	ASD	532,259	48,167	15,209
	ADHD	532,259	116,264	37,046
Pooled				
	ASD	985,444	75,222	24,516
	ADHD	985,444	206,145	68,991

*ADHD*, attention-deficit/hyperactivity disorder; *ASD*, autism spectrum disorder.

*Hegvik et al. Labor epidural analgesia and subsequent risk of offspring autism spectrum disorder and attention-deficit/hyperactivity disorder. Am J Obstet Gynecol 2023*.

## AJOG at a Glance

### Why was this study conducted?

A previous study has reported an association between labor epidural analgesia and offspring autism spectrum disorder (ASD), but the causal nature of this association remains unclear. It is possible that elevated depressive and anxiety symptoms in mothers who choose labor epidural analgesia are genetically associated with the ASD risk of their offspring and as a result may confound the association between labor epidural analgesia and offspring ASD risk.

### Key findings

In this multinational cohort study of 4,498,462 children using unexposed siblings as controls to account for shared familial risks (eg, genetic differences and early-life environments) in addition to measured individual-level confounders, no associations were found between labor epidural analgesia during delivery and offspring ASD or attention-deficit/hyperactivity disorder (ADHD).

### What does this add to what is known?

In this study using total population data derived from 3 different countries, we did not find support for the hypothesis that exposure to labor epidural analgesia causes an increased risk of either offspring ASD or ADHD. In contrast to previous replication efforts, the current study was able to explicitly account for shared familial risks while being sufficiently large to estimate the associations with a high degree of precision. As the decision to use epidural analgesia in labor is unlikely to cause offspring neurodevelopmental disorders, there is no need to revise the current clinical guidelines.

## References

[1] Anim-Somuah M, Smyth RMD, Cyna AM, Cuthbert A (2018). Epidural versus non-epidural or no analgesia for pain management in labour. Cochrane Database Syst Rev.

[2] Lim G, Facco FL, Nathan N, Waters JH, Wong CA, Eltzschig HK (2018). A review of the impact of obstetric anesthesia on maternal and neonatal outcomes. Anesthesiology.

[3] Hillyard SG, Bate TE, Corcoran TB, Paech MJ, O’Sullivan G (2011). Extending epidural analgesia for emergency caesarean section: a meta-analysis. Br J Anaesth.

[4] Seijmonsbergen-Schermers AE, van den Akker T, Rydahl E (2020). Variations in use of childbirth interventions in 13 high-income countries: a multinational cross-sectional study. PLoS Med.

[5] Ruppen W, Derry S, McQuay H, Moore RA (2006). Incidence of epidural hematoma, infection, and neurologic injury in obstetric patients with epidural analgesia/anesthesia. Anesthesiology.

[6] Pitkänen MT, Aromaa U, Cozanitis DA, Förster JG (2013). Serious complications associated with spinal and epidural anaesthesia in Finland from 2000 to 2009. Acta Anaesthesiol Scand.

[7] Qiu C, Lin JC, Shi JM (2020). Association between epidural analgesia during labor and risk of autism spectrum disorders in offspring. JAMA Pediatr.

[8] American Society of Anesthesiologists (2020). Labor epidurals do not cause autism; safe for mothers and infants, say anesthesiology, obstetrics, and pediatric medical societies.

[9] McKeen DM, Zaphiratos V, Canadian Anesthesiologists’ Society (2021). Lack of evidence that epidural pain relief during labour causes autism spectrum disorder: a position statement of the Canadian Anesthesiologists’ Society. Can J Anaesth.

[10] Royal College of Anaesthetists (2020). No evidence that labour epidurals cause autism.

[11] Kern-Goldberger AR, Burris HH, Levine LD (2021). Methodologic concerns with concluding a link between epidural and autism spectrum disorder. JAMA Pediatr.

[12] Lee A, Guglielminotti J, Landau R (2021). Methodologic concerns with concluding a link between epidural and autism spectrum disorder. JAMA Pediatr.

[13] Carrier FM, Lavoie A, Zaphiratos V (2021). Epidural analgesia during labour and autism risk: getting lost on the causal path. Can J Anaesth.

[14] Alder J, Fink N, Bitzer J, Hösli I, Holzgreve W (2007). Depression and anxiety during pregnancy: a risk factor for obstetric, fetal and neonatal outcome? A critical review of the literature. J Matern Fetal Neonatal Med.

[15] Smorti M, Ponti L, Tani F (2019). The effect of maternal depression and anxiety on labour and the well-being of the newborn. J Obstet Gynaecol.

[16] Meier SM, Deckert J (2019). Genetics of anxiety disorders. Curr Psychiatry Rep.

[17] McIntosh AM, Sullivan PF, Lewis CM (2019). Uncovering the genetic architecture of major depression. Neuron.

[18] Lichtenstein P, Carlström E, Råstam M, Gillberg C, Anckarsäter H (2010). The genetics of autism spectrum disorders and related neuropsychiatric disorders in childhood. Am J Psychiatry.

[19] Sandin S, Lichtenstein P, Kuja-Halkola R, Hultman C, Larsson H, Reichenberg A (2017). The heritability of autism spectrum disorder. JAMA.

[20] Bai D, Yip BHK, Windham GC (2019). Association of genetic and environmental factors with autism in a 5-country cohort. JAMA Psychiatry.

[21] Wang K, Gaitsch H, Poon H, Cox NJ, Rzhetsky A (2017). Classification of common human diseases derived from shared genetic and environmental determinants. Nat Genet.

[22] Grove J, Ripke S, Als TD (2019). Identification of common genetic risk variants for autism spectrum disorder. Nat Genet.

[23] Ghirardi L, Kuja-Halkola R, Butwicka A (2021). Familial and genetic associations between autism spectrum disorder and other neuro-developmental and psychiatric disorders. J Child Psychol Psychiatry.

[24] D’Onofrio BM, Sjölander A, Lahey BB, Lichtenstein P, Öberg AS (2020). Accounting for 233.e10 American Journal of Obstetrics & Gynecology FEBRUARY 2023 confounding in observational studies. Annu Rev Clin Psychol.

[25] Wall-Wieler E, Bateman BT, Hanlon-Dearman A, Roos LL, Butwick AJ (2021). Association of epidural labor analgesia with offspring risk of autism spectrum disorders. JAMA Pediatr.

[26] Hanley GE, Bickford C, Ip A (2021). Association of epidural analgesia during labor and delivery with autism spectrum disorder in offspring. JAMA.

[27] Mikkelsen AP, Greiber IK, Scheller NM, Lidegaard Ø (2021). Association of labor epidural analgesia with autism spectrum disorder in children. JAMA.

[28] Ren T, Zhang J, Yu Y (2022). Association of labour epidural analgesia with neuro-developmental disorders in offspring: a Danish population-based cohort study. Br J Anaesth.

[29] Hammad IA, Meeks H, Fraser A (2020). Risks of cause-specific mortality in offspring of pregnancies complicated by hypertensive disease of pregnancy. Am J Obstet Gynecol.

[30] Sjölander A, Frisell T, Öberg S (2022). Sibling comparison studies. Annu Rev Stat Appl.

[31] Faraone SV, Asherson P, Banaschewski T (2015). Attention-deficit/hyperactivity disorder. Nat Rev Dis Primers.

[32] Ghirardi L, Brikell I, Kuja-Halkola R (2018). The familial co-aggregation of ASD and ADHD: a register-based cohort study. Mol Psychiatry.

[33] Maret-Ouda J, Tao W, Wahlin K, Lagergren J (2017). Nordic registry-based cohort studies: possibilities and pitfalls when combining Nordic registry data. Scand J Public Health.

[34] Langhoff-Roos J, Krebs L, Klungsøyr K (2014). The Nordic medical birth registers e a potential goldmine for clinical research. Acta Obstet Gynecol Scand.

[35] Finnish Institute for Health and Welfare (2015). Trends in access to specialised health care.

[36] Bakken IJ, Ariansen AMS, Knudsen GP, Johansen KI, Vollset SE (2020). The Norwegian Patient Registry and the Norwegian Registry for Primary Health Care: research potential of two nation-wide health-care registries. Scand J Public Health.

[37] Ludvigsson JF, Andersson E, Ekbom A (2011). External review and validation of the Swedish national inpatient register. BMC Public Health.

[38] Furu K, Wettermark B, Andersen M, Martikainen JE, Almarsdottir AB, Sørensen HT (2010). The Nordic countries as a cohort for pharmacoepidemiological research. Basic Clin Pharmacol Toxicol.

[39] Lampi KM, Sourander A, Gissler M (2010). Brief report: validity of Finnish registry-based diagnoses of autism with the ADI-R. Acta Paediatr.

[40] Joelsson P, Chudal R, Gyllenberg D (2016). Demographic characteristics and psychiatric comorbidity of children and adolescents diagnosed with ADHD in specialized healthcare. Child Psychiatry Hum Dev.

[41] Surén P, Havdahl A, Øyen AS (2019). Diagnosing autism spectrum disorder among children in Norway. Tidsskr Nor Laegeforen.

[42] Idring S, Rai D (2012). Autism spectrum disorders in the Stockholm youth cohort: design, prevalence and validity. PLoS One.

[43] Larsson H, Rydén E, Boman M, Långström N, Lichtenstein P, Landén M (2013). Risk of bipolar disorder and schizophrenia in relatives of people with attention-deficit hyperactivity disorder. Br J Psychiatry.

[44] Wu CS, Nohr EA, Bech BH, Vestergaard M, Catov JM, Olsen J (2011). Diseases in children born to mothers with preeclampsia: a population-based sibling cohort study. Am J Obstet Gynecol.

[45] Viechtbauer W (2010). Conducting meta-analyses in R with the metafor package. J Stat Softw.

[46] Zhang T, Brander G, Mantel Ä (2021). Assessment of cesarean delivery and neuro-developmental and psychiatric disorders in the children of a population-based Swedish birth cohort. JAMA Netw Open.

[47] Oberg AS, D’Onofrio BM, Rickert ME (2016). Association of labor induction with offspring risk of autism spectrum disorders. JAMA Pediatr.

[48] Wiggs KK, Rickert ME, Hernandez-Diaz S (2017). A family-based study of the association between labor induction and offspring attention-deficit hyperactivity disorder and low academic achievement. Behav Genet.

[49] Ginsberg Y, D’Onofrio BM, Rickert ME (2019). Maternal infection requiring hospitalization during pregnancy and attention-deficit hyperactivity disorder in offspring: a quasi-experimental family-based study. J Child Psychol Psychiatry.

[50] Skoglund C, Chen Q, D’Onofrio BM, Lichtenstein P, Larsson H (2014). Familial confounding of the association between maternal smoking during pregnancy and ADHD in offspring. J Child Psychol Psychiatry.

[51] Kalkbrenner AE, Meier SM, Madley-Dowd P (2020). Familial confounding of the association between maternal smoking in pregnancy and autism spectrum disorder in offspring. Autism Res.

[52] Arrhenius B, Sariaslan A, Suominen A, Sourander A, Gyllenberg D (2021). Familial confounding affected the associations between maternal smoking during pregnancy and offspring speech and language, scholastic and coordination disorders. Acta Paediatr.

[53] American College of Obstetricians and Gynecologists’ Committee on Practice Bulletins— Obstetrics (2019). ACOG Practice Bulletin No. 209: obstetric analgesia and anesthesia. Obstet Gynecol.

[54] (2016). Practice guidelines for obstetric anesthesia: an updated report by the American society of anesthesiologists task force on obstetric anesthesia and the Society for Obstetric Anesthesia and Perinatology. Anesthesiology.

[55] National Institute for Health and Care Excellence (NICE) (2019). Intrapartum care for healthy women and babies (NICE Guideline CG190).

[56] D’Onofrio BM, Lahey BB, Turkheimer E, Lichtenstein P (2013). Critical need for family-based, quasi-experimental designs in integrating genetic and social science research. Am J Public Health.

[57] Larsson H, Sariaslan A, Långström N, D’Onofrio B, Lichtenstein P (2014). Family income in early childhood and subsequent attention deficit/hyperactivity disorder: a quasi-experimental study. J Child Psychol Psychiatry.

[58] Sariaslan A, Mikkonen J, Aaltonen M, Hiilamo H, Martikainen P, Fazel S (2021). No causal associations between childhood family income and subsequent psychiatric disorders, substance misuse and violent crime arrests: a nationwide Finnish study of >650 000 individuals and their siblings. Int J Epidemiol.

[59] Sariaslan A, Larsson H, D’Onofrio B, Långström N, Fazel S, Lichtenstein P (2015). Does population density and neighborhood deprivation predict schizophrenia? A nationwide Swedish family-based study of 2.4 million individuals. Schizophr Bull.

[60] Anorson N, Male I, Farr W, Memon A (2021). Prevalence of autism in Europe, North America and Oceania, 2000-2020: a systematic review. Eur J Public Health.

[61] Polanczyk GV, Salum GA, Sugaya LS, Caye A, Rohde LA (2015). Annual Research Review: a meta-analysis of the worldwide prevalence of mental disorders in children and adolescents. J Child Psychol Psychiatry.

