# Supplementary material for: Labor epidural analgesia and subsequent risk of offspring autism spectrum disorder and attention-deficit/hyperactivity disorder: a cross-national cohort study of 4.5 million individuals and their siblings
Source: Am J Obstet Gynecol. Author manuscript; Available in PMC 2025 Sep 8. (PMC7618080; doi:10.1016/j.ajog.2022.08.016)
Supplement: Supplemental Table [file EMS208256-supplement-Supplemental_Table.pdf]

SUPPLEMENTAL TABLE
Inclusion criteria for the nationwide samples

Criteria	Finland	Norway	Sweden	Pooled	Excluded
All individuals in the targeted cohorts	1,125,424	965,882	2,512,569	4,603,875	—
Could be linked to both biological parents	1,112,142	947,128	2,487,759	4,547,029	56,846
Not missing data on gestational age at birth	1,104,992	941,993	2,485,198	4,532,183	14,846
Not missing data on cesarean delivery	1,104,095	941,993	2,485,198	4,531,286	897
Did not migrate before age 1 y or 5 y[a]	1,099,580	931,408	2,478,915	4,509,903	21,383
Did not die before age 1 y or 5 y[a]	1,097,266	929,560	2,471,636	4,498,462	11,441

[a] The prescription drugs register started in 2004 in Norway when the oldest cohort were 5 years of age. The Norwegian National Patient Register had a later start in 2008. We therefore excluded all children who were right-censored before reaching the age of 5 years in the Norwegian sample.

Hegvik et al. Labor epidural analgesia and subsequent risk of offspring autism spectrum disorder and attention-deficit/hyperactivity disorder. Am J Obstet Gynecol 2023.
